# The impact of cold on the respiratory tract and its consequences to respiratory health

**DOI:** 10.1186/s13601-018-0208-9

**Published:** 2018-05-30

**Authors:** Maria D’Amato, Antonio Molino, Giovanna Calabrese, Lorenzo Cecchi, Isabella Annesi-Maesano, Gennaro D’Amato

**Affiliations:** 1 Respiratory Department, ‘Federico II University’ – Division of Respiratory Medicine and Allergy, Hospital Dei Colli, Naples, Italy; 2 Interdepartmental Center of Bioclimatology, University of Florence, Florence, Italy; 3 Epidemiology of Allergic and Respiratory DIseases Department, IPLESP, INSERM & Sorbonne Université, Medical School Saint-Antoine, Paris, France; 4 Department of Respiratory Diseases, High Specialty Hospital ‘A. Cardarelli’ and University of Naples Federico II, School of Specialization in Respiratory Diseases, Rione Sirignano, 10, 80121 Naples, Italy

**Keywords:** Bronchial asthma, Airway hyperreactivity in asthma and COPD, Cold induced respiratory diseases, Climate change, Global warming and health, Air conditioning and asthma and COPD

## Abstract

The increasing use, and sometimes the abuse, particularly in industrialized countries of air conditioning at home, in car, hotel and shopping centres has highlighted new emerging public health issues, resulting from exposure of the airways to cool air or, more properly, resulting from sudden temperature changes. This is part of a wider problem, relating to air quality in indoor environment, such as homes or offices, where people spend more than 90% of their time. In particular, if indoor exposure occurs quickly and without any gradual adaptation to a temperature 2°–3° lower than the external temperature and especially with a 5° difference (avoiding indoor temperature below 24°) and an humidity between 40 and 60%, there is a risk of negative consequences on the respiratory tract and the patient risks to be in a clinical condition characterized by an exacerbation of the respiratory symptoms of his chronic respiratory disease (asthma and COPD) within a few hours or days. Surprisingly, these effects of cold climate remain out of the focus of the media unless spells of unusually cold weather sweep through a local area or unstable weather conditions associated with extremely cold periods of increasing frequency and duration. Moreover, the energy consumed by air conditioning induces an increase of CO_2_ in atmosphere with increase of global warming. There is a need to better define the consequences of repeated exposure to cold air and the mechanisms by which such exposure could modify airway function and affect the outcomes of patients with pre-existing airway disease. This could help to promote adequate policy and public health actions to face the incoming challenges induced by climate change and global warming.

## Background

It is common knowledge that the winter season, especially in the higher latitudes, is the difficult part of year for patients with chronic respiratory diseases and that inhalation of cold air has negative effects on the lungs for people with respiratory diseases and in particular on asthma patients. However, surprisingly these effects of cold climate remain out of the focus of the media except in the case of unusually cold weather spells or unstable weather conditions associated with extremely cold periods of increasing frequency and duration.

During warmer months, cold air continues to be a problem with the overuse of air conditioning and a question is on the effects of its abuse, particularly when it is regulated at very cold temperature, which is a frequent event in some countries. The increasing use, in particular in industrialized countries, of air conditioners at home, in car, hotel and shopping centres has highlighted new emerging public health issues, resulting from exposure of the airways to cool air or, more properly, resulting from sudden temperature changes. This is part of a wider problem, relating to air quality in enclosed environments, in homes or offices, where people spend more than 90% of their time.

In recent years more discussion has taken place on “Indoor Air Quality” and more attention is being paid to related pathologies, from simple thermal discomfort to real pathologies such as sickness building syndrome [1] or aggravation of asthma and COPD.

The purpose of the present work is to better understand the consequences of repeated exposure to cold air by exploring the mechanisms by which such exposure could modify airway function and affect health outcomes of patients with pre-existing airway disease. In regards to health, we will describe the effects of cold air at first in healthy people like athletes and successively in respiratory patients. In regards to exposure, we will take into account the various risk factors interacting with cold temperature and air conditioning such as other meteorological variables, air pollution, biocontaminants and tobacco smoking, and their impact on respiratory health. The final aim of our work is to contribute to the promotion of adequate policy and public health actions to face the incoming challenges induced by climate change and global warming.

## Cold and air conditioning impact on respiratory health

Clinical discomfort due to respiratory illnesses can be exacerbated by indoor cold temperatures due to air conditioning. For chronic patients with precarious respiratory balances there is a risk of worsening of symptoms. Respiratory infections can be caused by cold air through increased bronchial inflammation caused by association of trigger factors such as cold and infections are both able to destabilize the patient.

Other trigger factors to consider, associated with cold and infectious agents, are cigarette smoke, urban pollution, inhalation of pollutants and irritants present in the air and in the working environments.

Patients with bronchial hyperreactivity are at risk of bronchospasm as a result of suddenly breathing cold air due to a variation in the inner balance of lower airways.

When air temperature drops quickly without any gradual adaptation, even for changes as low as 2°–3°, but especially for changes greater than 5°, there are possible negative consequences on their respiratory system and the patient is at risk of severe exacerbation of the symptoms of their obstructive respiratory disease (asthma and COPD).

Cold-induced airway damage is not only due to the direct effect of temperature, but also depends on the hyperventilation. Cooling of the airways is enhanced by increasing the airflow within the airways. Breathing of + 20 °C air at 15 l/min decreases the tracheal temperature to 34 °C whereas breathing similar air at 100 l/min decreases this temperature to 31 °C. Therefore, hyperpnea of temperate air shares similar effects to the inhalation of cold air [2].

Airways are lined by a thin layer of liquid, the airway surface fluid (ASL). Hyperpnea of cold air may cause the ASL to evaporate more rapidly than it can be replaced [3, 4], leading to drying and hypertonicity of the ASL. Of note, the absolute water content of subfreezing air is always near zero regardless of the level of saturation [5–7]. Therefore, while the effect of cold air on the skin is mainly cooling, the effect on the airways is both cooling and drying.

Under normal conditions, nasal breathing compensates in part for the effects of the cold air, and therefore, at rest and during light exercise the possible trigger sites for cold air- provoked respiratory symptoms include the facial skin and the nasal mucosa but not the lower airways.

The response mechanisms of airway inhalation of cold air go beyond changes of the ASL and involve a complex integrated system including the ASL but also mucosa, smooth muscle and blood vessels. Alveolar air, under normal conditions, is at a temperature of 37 °C and alveolar gas is fully saturated with water vapor at this temperature, properly humidified and heated by the components of the upper respiratory tract walls [8]. The role of these walls is not only to allow gaseous exchange, but it provides a large contact surface with the outside, it must also ensure adequate protection, in particular from dehydration and cooling. Inhalation of cold air induces activation of the epithelium to generate proinflammatory substances and that epithelial injury, determines activation of any exposed peripheral nerves. Vasomotor control in the airways is mediated by parasympathetic and sympathetic nerves, that through the release of neuropeptides such as Substance P and Calcitonin Gene Related Peptide (CGRP) [9], which can induce powerful vasodilation. Substances used for inhalation tests such as histamine, methacholine or substances locally released such as prostaglandins, produced locally by cells such as mast cells or eosinophils act on bronchial blood flow. Vasodilatation of bronchial vessels has been shown to cause thickening of the airway mucosa and should antagonize the effects of hyperventilation, but also helps to stimulate bronchial hyperresponsiveness that can trigger asthma attacks in predisposed subjects.

## Mechanisms of cold air effects in athletes

The respiratory system may be particularly affected by cold air exposure as inspired air has to be conditioned before participating in peripheral lung gas exchange, with an associated loss of heat and water. During exercise, a shift from nose to combined nose-and-mouth breathing takes place when the ventilation level exceeds approximately 30 l/min [7]. In such conditions, the possible trigger sites provoking respiratory symptoms include nasal mucosa, pharynx, larynx and the lower airways [6].

During physical exercises, nasal breathing quickly switches to mouth breathing, particularly at minute ventilations above 40 l/min, with the involvement of intrathoracic airways in this conditioning process [10].

Although exercising in cold air has minimal influence on the airways of normal individuals, it can induce a bronchoconstriction in asthmatic subjects and worsen airway obstruction in those with obstructive pulmonary diseases [11–13]. Winter athletes can be particularly affected by these environmental conditions, and an increased prevalence of airway hyperresponsiveness, asthma and chronic cough has been described in this population [14–18]. Bronchial biopsies of winter athletes have shown evidence of airway remodelling, possibly due to repeated cold-air and hyperventilation damage to the airways, although more research is needed on this influence on airway function [19, 20]. The mechanism of bronchoconstriction as a response to exercise-induced hyperpnoea, particularly in cold air, has been studied and appears primarily related to an increase in airway fluid osmolarity following hyperpnoea, although heat loss may be a modulator of this response, as well as a possible post-exercise “rewarming” of the airways [21].

Even in subjects without respiratory diseases, cold air can induce changes in the airways. Exposure to cold air can increase the number of granulocytes and macrophages in the lower airways [22]. Furthermore, cold-related impairment of respiratory mucociliary function can inhibit the clearance of pollutants [23]. Finally, in extreme cold temperatures, people tend to gather indoors and crowding can promote the transmission of infectious agents with ensuing airway inflammatory events.

Repeated cooling and drying of the airways are likely to take place in endurance athletes who frequently exercise at elevated ventilation levels. Indeed, a high prevalence of respiratory symptoms and airway hyperresponsiveness has been found in skiers, swimmers and long-distance runners. Studying the inflammatory infiltrate of the mucosa of the athletes with long and repeated exposure to cold air, identified a cell population different from asthma, with a greater number of neutrophils and a lesser number of eosinophils, mast cells and macrophages [22]: this further confirms that asthma and cold related diseases are two different entities, which, however, can influence each other.

## Cold air alone or in combination with other factors

### Cold and meteorological variables

The effect of cold temperature is modulated by other ambient conditions, too. As an example, cold damp air was reported by asthmatic patients to cause more symptoms than cold dry air, while a control group reported very few respiratory symptoms [11]. However, we need to consider combinations of several meteorological variables able to act on airways. Such combinations are referred to as “synoptic air masses”, where humidity, visibility, cloud cover, air pressure, wind speed and others are added into the equation and are known to influence mortality and morbidity [24–26].

### Cold and air pollution

Climate change and air pollution due to anthropogenic activities are intrinsically connected with many greenhouse gases and particulate air pollutants originate from the same source, such as fossil fuel combustion [1].

Nitrate particles and organic carbon aerosols have a cooling effect on the climate. Sulfur dioxide partly converts to sulfate particles, which also have cooling potential, so they partly react with black carbon, neutralizing its strong warming effect [27].

Some studies, including the paper of Carder et al. [28] highlight that cold temperature in conjunction with black smoke concentrations increase respiratory mortality. Since extremes of cold and particulate pollution may coexist, for example during temperature inversion during winter, these results may have important public health implications. Cold is related to various acute or long term airways diseases. General exposure to cold exacerbates chronic bronchitis and triggers Raynaud’s phenomenon of the lung (constriction of the pulmonary arteries and reduction of pulmonary blood volume in subjects with primary Raynaud phenomenon). Moreover, breathing very cold air at very high ventilation levels can led to acute pulmonary oedema or to frozen lungs [8]. It was also shown that inhalation of cold air causes vasodilation and thus increasing blood flow to the central airways in contrast to vasoconstriction in the intraparenchymal area [29]. In subjects who repeatedly hyperventilate very cold air, repeated episodes of significant variations in bronchial blood flow can lead to alterations in walls of bronchi and of pulmonary arteries, leading to faster than average decrease of lung function and increased thickness of walls of pulmonary arteries (Eskimo lung) [29]. According to a more recent classification, it is possible to classify cold- related diseases of the airways into three types: the short term responses are those that develop within minutes in response to sudden cooling of the airways, subjects with asthma or rhinitis are especially prone to these response; the long-term responses are those that develop in response to repeated and longstanding cooling and drying of the airway, usually in endurance athletes; finally, there are the physiological, reflex-mediated lower airway responses to cooling of the skin or upper airways [2].

There is no “universal” numerical value of air temperature that can be accepted as cut-off point for “cold”. It is rather the magnitude of downward temperature change below the mean seasonal range for a given area that challenges the adaptive ability of people. Mortality increased to a greater extent with given fall of temperature in regions with warm winters, in populations with cooler homes, and among people who wore fewer clothes and were less active outdoors. As adaptive capacity shrinks with age, it is the elderly who are mostly affected. Thus, it has been documented that cold temperatures are associated with a 3–4% increase in daily mortality and hospitalization for respiratory causes in the population over 75 years old for each degree Celsius decrease in minimum temperature or minimum apparent temperature (defined as a combined indicator of temperature and humidity above a city specific threshold level ranging from 23 to 29 °C) [30].

To obtain information on the extent and severity of asthmatic symptoms during daily life in winter, a simple questionnaire was sent to 57 asthmatic patients and a control group of 180 age-matched men and women in Göteborg (Sweden), where the average winter temperature is at about the freezing point. About two-thirds of the asthmatic patients reported cold to be a factor causing breathing difficulties. In 37%, these symptoms made the patients avoid going out during the winter [11].

### Air conditioning, cold and cigarette smoke

Pathophysiological aspects in which repeated exposure to cold stimuli determines anatomical and functional alterations of the respiratory tract have been also discussed in the literature [1]. Cold air, that is temporarily inhaled, induces excessive secretions of airway mucus and elicits ciliary ultrastructural anomalies. The only exposure to cold stimuli, can defect, turn on the cold-mediated activation of the TRPM8 channel and determine mucus hypersecretion with excessive MUC5AC secretion, and obstacle mucociliary clearance through numerical and structural anomalies of the ciliary apparatus. The inhalation of cold air, however, can only activate the TRPM8 receptor, but cannot determine the baseline overexpression of this receptor in patients with COPD. Probably cigarette smoke, that is also the main risk factor for COPD, is the etiological factor for the elevated expression of the TRPM8 channel in these patients, and so they are predisposed and hypersensitive to cold stimulus, even as air conditioning [30]. The synergistic effect between smoking and exposure to cold air was also observed in other studies, evaluating changes in impedance in the respiratory tract after exposure to cold in young smokers and nonsmokers: a broncho-constricting effect extending largely into the small peripheral airways can be demonstrated by impedance measurement in a group of asymptomatic young smokers which is not observed in normal subjects after cold-air challenge [31, 32].

## Role of the upper airways in health and asthma

Breathing cold air has been long recognized to trigger bronchoconstriction in asthmatics. In a classical experiment Shturman-Ellstein et al. [33] demonstrated that if subjects with asthma breathed only through the nose during the exercise challenge, an almost complete inhibition of the post exercise bronchoconstrictive airway response was observed [33, 34]. However, as the nose is serving as outermost filter for the inspired air, it is exposed to environmental hazards with consequent high frequency of morbidity. Adding to the atopic predisposition, it is likely that asthmatic subjects have concomitant rhinitis, which does not allow proper conditioning of the inspired air with negative impact on the asthmatic condition. The cross-talk and interplay between upper and lower airways has been a center point in the philosophy of the Allergic Rhinitis and its Impact on Asthma (ARIA) initiative and has been reconfirmed over the years [35, 36].

The upper airways mucosal structures are particularly sensitive to cold air influences. Challenges with cold dry air have been proposed to assess the state of nasal responsiveness in both allergic and non-allergic rhinitis. This line of research is substantiating the importance of cold weather as trigger in the pathogenesis of rhinitis, which in turn is a recognized risk factor for the development of asthma. Cold weather spells as a characteristic feature of changing climate will need to be considered in assessing the risk for asthma, especially since heterogeneous human populations may adapt differently to them [36].

The microclimate refers to the complex temperature parameters, relative humidity, and air velocity, which affect the heat exchange between the individual and the environment. The values of these parameters must be maintained within very narrow ranges to maintain the ideal environmental conditions so that the subject can perceive so-called thermal well-being [1]. In this context it is necessary that all the parameters of the microclimate are appropriately adjusted: The human body is equipped with sophisticated thermoregulatory systems which, however, can be altered by environmental conditions [1].

When it is too hot, the thermoregulation system triggers a number of mechanisms that can deliver heat to the outside, while when it is too cold, it works by limiting the heat dispersion. Microclimate can affect heat exchanges between individuals and the environment and in some situations hinder the thermoregulation mechanisms. For example, high humidity values in the summer can increase the heat-related discomfort: the high presence of water vapor in the air hampers the evaporation of the water contained in the sweat, which is the fundamental process for the human body to disperse excess heat. This explains why, in the presence of sultriness, a climatic situation characterized by a high relative humidity value, the human body tolerates less heat discomfort and the perceived temperature than the actual ambient temperature. The reason why the wind can increase the discomfort associated with a cold feeling is related to the fact that it increases the rate at which the body loses heat. The so-called perceived temperature, that is, the feeling of “hot” or “cold”, is therefore tied not only to the actual temperature but also to the other environmental conditions [1].

In buildings with natural ventilation, the outside air penetrates through existing openings in the building enclosure, such as joints or cracks in the walls, intersect around the doors (infiltration) and through the opening of doors and windows. The outside air can be introduced in a closed environment through mechanical, or forced, ventilation system that can also perform the functions of heating or cooling the air inlet, depending on the season (thermal ventilation systems).

In recent years, driven by economic and environmental motivations, thermally insulated buildings, where indoor climate conditions are closely regulated by ventilation and air conditioning systems are most frequently built. Nevertheless, in many countries there are no rigid rules to regulate the construction of ventilation systems, and although there are many studies on the possibility of using sensors within indoor environments [6, 37], there are no defined values and no environments closed monitoring systems [37].

The Sick Building Syndrome (SBS): indicates a well-defined symptomatic picture, manifested in a large number of occupants of modern or recently renovated buildings, equipped with mechanical ventilation and global air conditioning systems (without supplying fresh air from the outside) and used in offices, schools, hospitals, homes for seniors, civilian homes; a still unknown, probably multifactorial etiology, linked to factors related to buildings, air conditioning and ventilation systems, maintenance programs, type and organization of work and personal factors [38].

Regarding the pathologies more specifically associated with the use of conditioning systems, they may be related to the failure to achieve microclimatic targets or because they can be dangerous sources of biological or chemical pollution, especially if they are badly designed, in poor state of cleaning and maintenance [39]. Nasal breathing of cold air induces an engorgement of the venous sinuses in the submucosa [5, 10], which leads to congestion, sneezing and, especially, rhinorrhea both in healthy and rhinitic subjects [13]. However, these responses are greater in subjects with rhinitis than in healthy subjects [40] and greater in subjects with asthma and rhinitis than in subjects with rhinitis alone [41]. Yet in a short time, cold air hyperpnea provokes bronchoconstriction in asthmatic subjects [42], especially in children and young adults [43, 44]. The pathophysiological mechanism beyond this response has been a matter of considerable debate: studies on the effect of cooling on the airways smooth muscle have been conflicting results [45–48]. Certain lower airway sensory receptors can be sensitive to cold and capable of inducing bronchoconstriction in animals [49, 50]. A fundamental role is certainly played by vasoconstriction, as already described before. It does not seem to be involved in a response mediated by eosinophils [51]. Besides bronchoconstriction, cold air hyperventilation also provokes coughing in susceptible people. Coughing and bronchoconstriction seem to be independent responses since pre-treatment with salbutamol blocks cold air-provoked bronchoconstriction but has no effect on cold air provoked coughing [52].

The long-term responses to cold exposure, include all those airways alterations, also anatomical, in part already described previously, comprising an increase in bronchoalveolar lavage fluid granulocytes in healthy humans [22], loss of ciliated epithelium, thickening of the lamina propria with increased concentrations of inflammatory cells, hyperresponsiveness and airway obstruction [53–57].

The last group finally includes reflex bronchoconstriction due to cold trigger of the skin or upper airway. It seems that the reflex bronchoconstriction provoked by facial or upper airway cooling is too mild to cause breathing difficulties in a person with near normal lung function. However, for a subject with severely impaired lung function these responses may be of clinical significance [58].

We do not know much about the molecular mechanisms underlying respiratory cold-related symptoms, but a role appears played by the receptor TRPM8. The discovery of thermosensitive ion channels of the transient receptor potential (TRP) family has demonstrated an underlying molecular mechanism for temperature detection. Transient receptor potential melastatin 8 (TRPM8) is a non-selective calcium permeable cation channel, that seems overexpressed and upregulated on the epithelium of patients with chronic lung disease and therefore, probably, is involved on hypersensitivity of this population to cold-related triggers [59] in association with phosphorylation of MARCKS-PSD [60].

## Air conditioning and respiratory infections

Exposure to air conditioners with very cold air, induces alterations of the respiratory airways that, mostly with pre-existing respiratory conditions such as asthma and COPD, may form a susceptible group, also in young adults [14], which not only can determine cold-related symptoms, as shortness of breath, wheezing, phlegm production, but also a greater susceptibility to infections. Indoor air can be an important vehicle for a variety of human pathogens airborne spread, already in normal conditions, as vegetative bacteria (staphylococci and legionellae), fungi (*Aspergillus*, *Penicillium*, and *Cladosporium* spp and *Stachybotryschartarum*), enteric viruses (noro- and rotaviruses), respiratory viruses (influenza and coronaviruses), mycobacteria (tuberculous and nontuberculous), and bacterial spore formers (*Clostridium difficile* and *Bacillus anthracis*) which can have pathogenic action on human health, together with exposure to other agents as noxious chemicals, particulates, pollen and other allergens [61]. Because these agents can infect a susceptible host, they must survive the prevailing environmental conditions, determined by air temperature, relative humidity (RH), turbulence, that are just a few of the factors involved, since a generalization is difficult considering the biological diversity of microorganisms. So it is obvious the role played by air conditioning, cold or warm. Various studies have shown that among the viruses, for example, rotavirus survived best at midrange RH but not at high temperature; among bacteria, staphylococci has ability to survive over a wide range of temperatures, RH, and exposure to sunlight [61].

As ubiquitous microorganisms, fungi pose a health threat in indoor environments. Fungal infections can be particularly serious in immunocompromised patients, especially airborne spores of *Aspergillus* spp that are blown in from natural ventilation sources. Fungal spores are aerosolized from municipal water supplies and dust and can be effectively transported over long distances by wind and air currents. The evolution of the fungal spore has enabled them to travel long distances and be more capable of withstanding environmental insults. The most important factor for fungal growth in indoor environments is humidity. In fact, results of many studies, showed that airborne fungal concentrations were not correlated to the diseases or personnel density, but were related to seasons, temperature, and relative humidity. There were similar dominant genera in all wards. They were *Aspergillus* spp *Penicillium* spp and *Alternaria* spp. Therefore, attention should be paid to improve the filtration efficiency of particle size of 1.1-4.7 μm for air conditioning system of wards. It also should be targeted to choose appropriate antibacterial methods and equipment for daily hygiene and air conditioning system operation management [62]. In fact, the risk of air conditioning-caused infections is increased when, to save on air cooling costs, especially with regard to the air conditioning of rooms with large volumes of air to cool (department stores, ships, airplanes, etc.), instead of cooling hot air coming from the outside, it is preferable to keep the air cooled previously cooled from the inside (recirculation function) cool. This, however, significantly reduces air exchange in the environments while increasing the concentration of pollutants (irritants, fumes, allergenic pollens, etc.) and infectious agents (viruses and bacteria) that can add to their pathogenic activity already in itself represented by the particular physical characteristics of an artificially cold and dry air. Much work is directed at the need to use air filters for the control of respiratory diseases, especially of an allergic type, by applying filtering systems that regulate the level of pollution [63].

## Conclusions

There is a need to better define the consequences of repeated exposure to cold air and the mechanisms by which such exposure could modify airway function and affect the outcomes of patients with pre-existing airway disease [1]. This could help to promote adequate policy and public health actions to face the incoming challenges. By all means distinction should be drawn between effects on individuals and effects on populations, as populations are heterogeneous in their susceptibility, for example, a different response to cold exposure was studied by race [63], but reversible and irreversible effects should be identified.
